# Media Coverage of Alcohol Issues: A Critical Political Economy Framework—A Case Study from Ireland

**DOI:** 10.3390/ijerph14060650

**Published:** 2017-06-16

**Authors:** Julien Mercille

**Affiliations:** School of Geography, University College Dublin, Belfield, Dublin 4, Ireland; julien.mercille@ucd.ie; Tel.: +353-1-716-8382

**Keywords:** media, alcohol policy, political economy, minimum unit pricing, Ireland

## Abstract

There is a growing literature on news media representations of alcohol-related issues. However, current scholarship has neglected critical political economic frameworks to interpret media coverage of alcohol. This paper presents such a framework that conceives of news organisations as corporations that share the values and interests of political and economic elites. The media are thus expected to present viewpoints that are more aligned with the alcohol industry than the scientific consensus on public health policy would warrant. The media are also expected, but to a lesser extent, to present a certain amount of support for public health perspectives because these are supported by a few socioeconomic elite groups (the medical professions, progressive politicians). The case of Ireland from 2012 to 2017 illustrates the framework empirically. Four main newspapers’ coverage of the Public Health (Alcohol) Bill and related policies is examined. Results show that, overall, 44.0% of articles support public health measures and 56.0% are opposed or remain neutral. It is argued that the media are not strong proponents of public health for multiple reasons: there are more articles opposed to or neutral toward public health measures than supporting them; the number of supportive articles remains relatively small and there are still many pieces presenting drinks industry views; there are virtually no calls in the media for stronger measures; supportive coverage is partially explained by the pub owners lobby’s support for minimum unit pricing; the media often downplay or ignore the negative consequences of alcohol, such as its role in accidents; many news articles normalise drinking and promote events sponsored by the industry; there is not a single Irish journalist covering alcohol issues systematically; and other policy issues that are prioritised by elites receive multiple times more media coverage than public health measures. In short, the media reflect the views of the political and economic establishment on public health measures: there is some support from the medical professions and progressive politicians, but overall, there is a clear reluctance to support strong public health strategies. One main recommendation for public health advocates to promote their perspectives is to diversify the mass media and make them less commercial in nature, as well as to engage with non-corporate, so-called progressive “alternative media” outlets.

## 1. Introduction

Representations of alcohol in the media have received significant scholarly attention that has focused on advertising [1] and non-commercial media representations of alcohol in film [2], television, and music [3,4]. However, this paper investigates how alcohol issues are covered in the news media by examining newspapers. It engages with the growing literature on this subject, which has investigated news media coverage of minimum pricing [5,6,7,8], alcohol use [9], the alcohol industry itself [10], alcohol policy and education [11,12], alcohol advertising [13], alcohol in ethnic and racial minority newspapers [14], as well as specific pieces of alcohol-related legislation [15].

It is argued that although this scholarship has produced important insights, it could be strengthened by using a critical political economy framework to conceptualise the media. This would improve our understanding of news media representations of alcohol, and suggests novel ways to increase attention to public health perspectives in media reporting.

Existing analytical and methodological frameworks have neglected critical political economy (e.g., [16]). This is paradoxical, because many scholars have written about the role of the drinks industry in shaping alcohol policy debates [17,18,19]. Researchers are thus aware of the ways in which political economic power operates to shape public health policy and outcomes. Nevertheless, scholarship is virtually unanimous in using mainstream conceptualisations of the media that do not engage significantly with critical political economy. Mainstream approaches perceive news organisations as institutions that reflect and balance the range of viewpoints and interests in society. For example, in a representative statement, Törrönen [12] argues that “rather than indicating an autonomous force in society”, the media “reflect” changes in “policy and in public opinion”. Thus, news outlets are conceived as more or less disinterested conveyors of the range of views in society.

This is very different from the conceptualisation of the media presented in this paper, which conceives of news organisations as large businesses that are part and parcel of the corporate sector and close to ruling political circles. As a result, they share the interests and values of economic and political elites. This interpretation draws on the work of a number of theorists and political economists [20,21,22,23,24,25,26,27] and argues that three main factors explain why the media share establishment viewpoints and interests: corporate and government links, advertising pressures, and sourcing.

### 1.1. Corporate and Government Links

Mass media outlets are, first and foremost, corporations embedded in a capitalist economic system, of which they are integral parts. This has several consequences. First, to start and run a media company, significant financial resources are necessary, which means that only corporations and wealthy individuals are able to do so. As a result, a few media outlets usually dominate national airwaves and the print press, which reduces news diversity. Second, media firms are integrated into the market and they feel the pressures of shareholders, directors and bankers to generate profits. Relationships with economic and political elites are created and maintained through boards of directors and general business and social interactions. For instance, media organisations maintain close relationships with banks for loans, lines of credit, receiving advice, and service on the issuance and sale of bonds and shares, in addition to mergers and acquisitions. In particular, news outlets have acquired a range of other non-media and media firms while non-media businesses have acquired interests in media outlets. In short, media organisations have interests that are very similar to those of the corporate sector and thus the stories they present tend to reflect such interests and viewpoints. In fact, it would be surprising if news outlets emphasised stories critical of key values of the corporate world, like business rights, light regulation, and weak trade unions, as this would directly undermine their own position.

Third, the media also have a close relationship with the government, which has the capacity to influence news outlets to conform to state interests and views, which, in any case, are usually relatively aligned with that of the corporate sector. This is because news organisations depend on the state for franchises and licenses. The government is thus in a position to exert leverage over the orientation the media take in the news. More broadly, the media, like the business sector in general, depend on the state to maintain regulations favourable to private enterprise and a healthy business climate, at home and abroad if they wish to expand internationally.

### 1.2. Advertising Pressures

Advertising revenues are essential to today’s news industry. They allow newspapers to be sold for a cheaper price, making them more competitive. Media outlets unable to attract ads thus run the risk of bankruptcy. This affects news content because corporate advertisers tend not to subsidise news stories or television programmes that question seriously or attack their own business or the political economic system of which they are part. For example, a radio show on the economy sponsored by a bank is unlikely to repeatedly challenge the financial sector on fundamental issues for fear of losing its sponsor. The same goes for the drinks industry: it is unlikely to sponsor television and radio shows and newspapers strongly critical of the alcohol industry or that repeatedly present public health views.

### 1.3. Sourcing

Reporters depend on mainstream institutions to write their stories. Because of a competitive environment, time constraints, and limited resources, journalists must rely on institutions that provide a steady flow of news, which means large organisations that have the financial resources to produce regular press releases and hold press conferences. Corporations and the government are two such sources, resulting in their viewpoints being reported more predominantly in the media. Moreover, stories sourced to those organisations imply an image of credibility and objectivity. Regular sourcing interactions between journalists and powerful institutions result in close relationships between reporters and government and corporate officials. The latter can then deny privileged information to journalists who do not adopt the expected storylines.

### 1.4. Implications

I argue that a critical political economy framework has two main implications with regard to existing research. First, it suggests that contemporary mass media outlets have, by their institutional nature, a tendency to oppose public health approaches to alcohol and to be supportive of the alcohol industry. This is because media firms share the corporate values of the alcohol industry, receive vital advertising funding from drinks firms, and rely on their press releases and representatives as sources to write their stories. This does not mean, however, that media outlets are completely opposed to public health. Indeed, they reflect the range of elite interests and views. And because some socioeconomic elite groups are favourable to public health approaches, such as the medical professions and elements of the political establishment like centre-left political parties, their views do make it into the media, and sometimes significantly so. However, the alcohol and corporate lobbies, in addition to more conservative elements of the political establishment, have more power than the relatively smaller public health lobby.

It must be clarified that to say that the media tend to reflect industry viewpoints does not mean that such views necessarily account for more than 50% of stories or airtime on a given alcohol issue—often, they do not. The point is that even if fewer than 50% of stories accept a pro-alcohol industry view for which there is no or little scientific evidence, this still denotes a media biased toward the drinks industry, as stated by Katikireddi and Hilton [6]. In other words, even a modest amount of coverage for pro-industry views that have little basis in fact should be interpreted as bias.

Second, although public health advocates may engage with the mass media successfully to increase their exposure, there are limits to this strategy. This is because the corporate nature of the media is inherently tilted against public health. Indeed, it would be very unlikely that the mass media become directly reflective of the public health community views and discard industry views. However, in existing scholarship, many recommendations do not consider the obstacles posed by such institutional factors. These recommendations call on public health advocates to make greater efforts to build relationships with journalists to explain public health research to them [28,29]. However, from a critical political economy perspective, the main reason why journalists do not report enough on public health findings and conversely report a lot more than they should on industry views is not because they are naively unaware of public health research. Rather, it is because they work for media institutions that are reluctant to oppose alcohol firms and therefore tend not to direct their journalists towards the critical investigation of alcohol issues. This suggests that in order to maximise media exposure for public health advocates, the mass media would need to be restructured. News outlets should be reorganised along lines that are more diversified, less corporate, and less commercial. Media that do not share corporate values and that are not dependent on the corporate sector (including the drinks industry) for advertising revenues should not be pulled as much towards the interests of the alcohol industry. This is the case of the so-called progressive “alternative media”, which, although smaller than the mass media, should nevertheless be more open to publishing stories from a public health perspective. (To avoid confusion, it should be clarified that it is the progressive, left-of-centre alternative media that is considered here, not right-wing, jingoist, or “fake news” alternative media outlets).

### 1.5. The Case of Ireland

The theoretical framework outlined above is applied to the media coverage of Ireland’s Public Health (Alcohol) Bill and related alcohol issues. As of this writing, the Bill has not been enacted by Parliament due to the opposition of the alcohol industry and significant elements within the government. The Bill originated from recommendations made in a 2012 report, the Steering Group Report on a National Substance Misuse Strategy [30]. However, since then, the government has moved very slowly on the Bill, pressurised by the drinks industry lobby and many political officials opposed to public health legislation [31]. This has been a recurring pattern in recent decades, as Ireland’s neoliberal political and economic establishment has successfully resisted the implementation of public health legislation [32,33,34,35,36]. Indeed, only in 2015 was the Bill’s general scheme finally published, but it may never be enacted [37]. The Bill’s two main measures that have generated public debate are minimum unit pricing and a ban on drinks sponsorship of sports and cultural events. The latter was in fact dropped from the Bill due to opposition from the alcohol industry and politicians, but minimum pricing is still included.

The government’s reluctance to enact the Bill is unfortunate, because Irish alcohol consumption levels remain high by international standards, as does the prevalence of negative consequences of such consumption. Consumption reached a high of 14.3 L of alcohol per adult in 2001 and dropped to 11.9 L in 2010 due to the economic recession that began in 2008 [30]. Excessive alcohol consumption and high levels of binge drinking also come at a high price economically. A study calculated that in 2007 the cost of harmful alcohol use was €3.7 billion or 1.9% of GNP (gross national product) [30]. In particular, costs to the health care system amounted to €1.2 billion and approximately a quarter of all injuries to emergency hospital admissions are alcohol related, while alcohol accounts for some 2000 beds occupied per night in acute hospitals [38].

In Ireland, the mass media conform to the model outlined above. Independent News & Media (INM) is the dominant news conglomerate and is listed on the Irish and London stock exchanges. It controls the *Irish Independent* and *Sunday Independent*, analysed below. A survey reported that it owns 200 newspapers and magazines and 130 radio stations in Ireland, the United Kingdom, and several other countries [39]. It is controlled by the prominent Irish businessman Denis O’Brien, a mobile phone tycoon who has a net worth estimated at $4.8 billion, number three on the Irish rich list [40].

The *Irish Times*, also analysed below, is Ireland’s newspaper of record. Its board has included a number of establishment figures, such as David Went, former director of Goldman Sachs Bank (Europe) and CEO of financial firm Irish Life & Permanent; Terence O’Rourke, of Enterprise Ireland, Hibernia REIT (a large property investment vehicle), and formerly of global auditing firm KPMG; Brian Caulfield, a venture capitalist; and Eoin O’Driscoll, who has been a member of the National Executive Council of IBEC, Ireland’ s largest business and employers’ lobbying organisation, and on the board of Barclay’s Bank Ireland [41].

The *Irish Daily Mail*, a tabloid analysed below, is owned by Jonathan Harmsworth, the British aristocrat formerly known as the Viscount Rothermere, who inherited the newspaper from his father. He has a net worth of $1.2 billion [42]. Its board members are embedded in the corporate world and include Kevin Parry (KPMG Managing Partner), Dominique Trempont (various investment and technology firms), and Jo Roizen (corporate executive and venture capitalist), among others [43].

There is no precise data on alcohol-related advertising in the Irish media, but an oft-cited figure is that the annual advertising spent by the industry is €24 million, in addition to about a quarter of the €67 million spent by supermarkets in the media, which includes alcohol products [44]. Whatever the exact sum that makes it into newspapers, it is valuable to media outlets. There are supermarket advertising supplements in many daily newspapers, which virtually all contain alcohol products, and there are direct ads placed in newspapers and on television. There is no doubt that this influences news content to be more favourable to the alcohol industry, although the precise strength of the influence is difficult to measure. As a proxy, the impact of property (real estate) advertising on news content in Irish newspapers has been studied more thoroughly. For example, weekly property supplements in newspapers have led to favourable news coverage of the housing boom in the early 2000s, and contributed to inflate the unsustainable bubble that collapsed in the years after 2007 [45]. An Irish journalist interviewed for an academic study stated that reporters “were leaned on by their organisations not to talk down the banks (and the) property market because those organisations have a heavy reliance on property advertising” [46]. Moreover, in testimony at the Irish Parliament’s Banking Inquiry, Geraldine Kennedy, *Irish Times* editor during the housing bubble years, stated that many telephone calls were made to the newspaper’s management office about news coverage and that some individuals in the property sector threatened that the *Irish Times* would never get an advertisement again after an article was published that predicted a collapse of the real estate market [47].

## 2. Methods

### 2.1. Newspapers Selection

The following newspapers were selected for analysis: *Irish Times*, *Irish Independent*/*Sunday Independent* and *Irish Daily Mail*. The *Irish Times* is Ireland’s newspaper of record, a quality paper with a readership of 427,000 [48]. It is published on weekdays and Saturdays. Its editorial stance is centrist overall, liberal on social issues but centre-right on economic issues. The *Irish Independent* and *Sunday Independent* are Ireland’s most popular newspapers, with readerships of 688,000 and 921,000, respectively [48]. Their style is right-wing populist. They are not considered tabloids but cannot be considered quality papers either. The *Irish Daily Mail* is a tabloid, the Irish edition of Britain’s *Daily Mail*. Its readership is 217,000 and it is published on weekdays and Saturdays [48]. Its editorial position is right-wing populist.

The newspapers selected cover the range of types of news outlets in Ireland (quality vs. tabloid) as well as the ideological spectrum. There are no left-of-centre newspapers in the country, only centrist and right-of-centre ones. The *Irish Times* and *Irish Independent*/*Sunday Independent* are the most important newspapers in terms of readership and influence. The *Irish Daily Mail* was selected because it is representative of the tabloids available in Ireland, and its articles are available in the LexisNexis database, just like the other selected papers.

### 2.2. Database Searches

The LexisNexis database was used to examine newspapers’ coverage of several alcohol-related issues. In order to obtain a representative and relevant sample, all searches were conducted for the time period from 1 January 2012 to 7 April 2017. The Steering Group report [30], from which emerged the Public Health (Alcohol) Bill, was published in February 2012 and therefore it makes sense to examine press coverage starting in 2012 up to the present (the database searches were conducted in April 2017). Public discussion of issues such as minimum unit pricing and drinks sponsorship predates 2012, but it is fair to start the analysis in 2012 to make it relevant to the current situation while providing depth by surveying a little over five years of media coverage.

All searches were restricted to articles discussing topics in the Irish context. For example, only articles related to minimum unit pricing in Ireland were included. Letters to the editor were removed from all searches as well as duplicate articles. Pieces of any length were included because even short ones can have as much, or even more, impact on public perceptions of alcohol issues. Key words were searched “at the start” of the articles (a formal LexisNexis command), that is to say, in articles’ headline and first few paragraphs. This methodology targets articles that directly discuss a topic (mention of a topic toward the end of an article usually means that it is not directly concerned with this subject and its inclusion in the analysis would thus be questionable). An alternative methodology would have been to include only articles that mention a key word, say, at least three times, but this would have excluded relevant pieces that mention a key word only once in their headline, for example, which obviously would be important, being directly concerned with the subject.

Four searches were conducted:

- **Search #1**: Public Health (Alcohol) Bill
  The exact search is:
  “public health (alcohol) bill” OR “alcohol bill” AT THE START
  This examines coverage of the Bill as a whole. Articles were classified into those favourable to the Bill, opposed to it, or neutral. Opinion articles and editorials are usually easy to classify because they present a clear viewpoint. News articles tend to be more neutral in tone; their classification depended on which viewpoints they voiced most prominently. For example, a news article outlining the views of a representative from the drinks industry opposed to the Bill would be classified as opposed to the Bill.
- **Search #2**: Minimum unit pricing
  The exact search is:
  (“minimum unit pricing” OR “minimum pric!”) AND alcohol AT THE START
  This examines coverage of the policy of minimum unit pricing of alcohol products (which is included in the Bill). Articles were classified into those favourable to the policy, opposed to it, or neutral, in the same way as described for Search #1. (The ! is the truncation symbol).
- **Search #3**: Drink industry sponsorship
  The exact search is:
  sponsor! AND (alcohol OR drink!) AT THE START
  This examines coverage of the proposed ban on drinks industry sponsorship of sports and cultural events, which was eventually dropped from the Bill. Articles were classified into those favourable to the ban, opposed to it, or neutral, in the same way as described for Search #1.
- **Search #4**: Guinness
  The exact search is:
  Guinness AT THE START
  This search provides one example of the ways in which the media present alcohol products beyond specific policy issues. There are many possible searches that could have been employed, but a simple one for mentions of Guinness makes the point. Guinness is a stout brewed by Diageo and is Ireland’s national drink, deeply associated with national identity [49]. The aim of this search is to examine whether products like Guinness have become normalised in public discourse, or if instead they generate significant opposition. Therefore, articles were categorised into whether they voiced a negative opinion of Guinness or not (it is often too difficult to separate neutral from positive articles that make only brief mentions of Guinness). The articles were assessed in the same way as described for Search #1. A low percentage of negative articles about Guinness would indicate that the product has become normalised.

## 3. Results

Table 1 presents the results of the database searches on the Bill and related policies. First, between 2012 and 2017, a total of 41 articles appeared in the four newspapers on the Public Health (Alcohol) Bill. Nearly half (46.3%) support it, while 19.5% oppose it and 34.1% are neutral. Second, 60.8% of pieces support minimum unit pricing and 10.8% oppose it. As many as 73.3% of *Irish Times* pieces support the strategy and only 3.3% oppose it. Third, the proposal to ban drinks industry sponsorship of sports and cultural events reveals a more divided media. More than a third (35.1%) of articles support the ban while 27.8% oppose it.

Overall, 44.0% of articles returned by the three searches support public health measures (the Bill itself, minimum unit pricing or the sponsorship ban), 21.8% are opposed, and 34.2% are neutral. Another way to put it is that while 44.0% of articles are supportive of public health measures, 56.0% do not voice support for them or are opposed to them.

Supportive articles note the significant public health problems caused by alcohol in Ireland and the benefits of restrictions on the drinks industry. However, relatively few pieces can be considered to be of good quality and to outline systematically relevant facts about alcohol problems in Ireland and to report research on public health. Indeed, very few articles are written by specialists or organisations promoting public health approaches in Ireland: a total of only five articles are penned by officials from the two main national organisations for public health in alcohol-related issues, Alcohol Action Ireland and the Royal College of Physicians of Ireland. These clearly explain why the Bill should be passed from a public health perspective. Many of the other favourable articles are written by journalists and tend to omit important facts or details. In particular, many do not make a strong case in favour of the Bill and related policies, which diminishes their capacity to influence readers.

On the opposition side, the main arguments deployed involve a rejection of the “nanny state”, perceived to be encroaching on individuals’ freedom by enacting public health laws. Other views simply downplay the scale of alcohol-related problems in Ireland. Still others argue that businesses would face increased costs to meet the new legislation’s requirements, which would hurt the economy. The strongest opposition is voiced against the proposed ban on drinks industry sponsorship. The main argument is that a ban would cut funding to sports and cultural events and jeopardise their existence. The interests of the alcohol industry and sporting and cultural bodies coincide and are given relatively ample space in newspapers. Therefore, even if in general, the media produce pieces favourable to public health legislation, alcohol industry voices are given a prominent voice, either through representatives writing op-eds, when journalists quote business representatives, or when news stories simply report industry views without direct attribution.

Table 2 presents the results of the search for articles mentioning Guinness in the headline or lead paragraphs. All newspapers include a large number of such pieces, for a total of 1889, in sharp contrast to the media coverage of alcohol policy issues (Table 1).

Pieces mentioning Guinness include cooking recipes, stories about the Guinness family, economic articles reporting on financial performance of Diageo (which makes Guinness), and references to drinking pints of Guinness in Irish culture. A large number of articles (more than half) mention events sponsored by Guinness, such as the Guinness Galway horse races, the Guinness Cork Jazz Festival, as well as the Guinness Pro12 rugby competition. These articles amount to advertisements for the brand.

The discussion section below argues that this is conducive to the normalisation of alcohol products by the media. Indeed, only a very small percentage (approximately 2%) of articles mentioning Guinness are negative, with the rest either positive or neutral. The negative pieces mostly criticise alcohol-problems in Ireland, and celebrations like Arthur’s Day, a global promotional event created by Guinness in reference to Arthur Guinness, the founder of the Guinness brewery.

## 4. Discussion

The critical political economic framework outlined above suggests that the mass media tend to share the values and interests of the political and economic establishment, which include those of the alcohol industry. Of course, there is diversity within elite circles and the views of the public health lobby and progressive politicians are expected to appear in news reports, but on balance, the media are expected to be more aligned with the alcohol industry than a public health perspective would warrant.

This section argues that Ireland’s case fits this interpretation. The Irish media may be described, at best, as reluctant supporters of public health measures, and perhaps more accurately, as rather opposed to such measures. This, in fact, is exactly how academic specialists have described the position of the Irish government and drinks industry toward alcohol [31,32,33,34,37].

There is support in the media for public health measures, with, overall, 44.0% of articles favourable against 56.0% that do not voice support (neutral, 34.2%) or are opposed (21.8%). However, there are significant reasons why the mass media should not be seen as supporters of public health as related to alcohol. First, the media coverage is much more favourable to the drinks industry than the evidence-based scientific consensus would warrant. Indeed, there is substantial agreement among public health experts that policies proposed in the Bill, minimum unit pricing and a sponsorship ban have positive effects on health [38,50]. However, in the Irish press, there are fewer articles in favour of such measures (44.0%) than pieces opposed to them or neutral (56.0%). This means that the majority of media coverage is not in line with the scientific consensus but rather supports the drinks industry by either denying the benefits of public health measures or remaining vague or ambiguous, which is exactly the approach used by the industry. Moreover, even if 50% of articles were in favour of the measures, this still would not denote a “balanced” media on alcohol issues. It would suggest bias against public health because scientific evidence would be downplayed [6]. A truly balanced media would reflect the scientific consensus, which is clearly in favour of the Bill. Yet, in the Irish media, there are still many articles denying its benefits, often repeating explicitly industry claims.

Second, the number of articles on alcohol policy issues is relatively small and the alcohol Bill is not a prominent issue in the Irish media. A few stories are written about it when legislative developments arise, but no more. For example, the *Irish Times*, Ireland’s newspaper of record, published only six pieces over more than five years directly supportive of the Bill, or about one per year. Even if one adds the articles favourable to minimum pricing and a sponsorship ban, the coverage remains minimal (and some articles were returned by more than one search, further reducing the total).

Third, although many articles are favourable to public health, very few call for stronger measures than those related to the alcohol Bill, which is relatively weak on public health measures (although it would still constitute a significant improvement on the current situation) [38]. For example, the Bill would restrict alcohol advertising on television to after 9 pm. However, virtually no piece makes the case for banning it completely, as stipulated in France’s alcohol laws. Neither does any article call to roll back the number of off-licenses in Ireland that sell cheap alcohol and that have mushroomed in recent years, their number growing by as much as 161% between 1998 and 2010 [30]. In other words, media coverage is either in favour of the Bill or in favour of something even weaker.

Fourth, media support for the Bill is partially explained by the fact that minimum pricing is, paradoxically, supported by a large segment of the alcohol industry, the vintners (the pub owners lobby). Pub owners like the measure because it does not affect the prices of alcohol in pubs, but it raises the price of cheap alcohol sold by the supermarkets, their direct competitors. Pubs are scattered all over Ireland geographically, and every politician is sensitive to local support. In other words, minimum unit pricing mobilises not only the public health lobby but also the vintners, who usually side with the alcohol industry. This, incidentally, illustrates that the alcohol industry is not always uniform and may be divided on some specific issues [51]. Favourable media coverage of minimum pricing is therefore due in part to the media conveying business views; it is no proof that news organisations are strong public health advocates.

Fifth, by contrast, when the establishment is united, it can block public health measures, which receive less support in the media, like the sponsorship ban, which was eventually dropped from the Bill. This is attributable to the power of the drinks industry to lobby against it, and some of their representatives have authored pieces in the press. The ban is also opposed by important sectors of the government, for two reasons. One, which applies to the Department of Transport, Tourism and Sport and the Department of Arts, Heritage and the Gaeltacht (Irish-speaking regions of Ireland) [37], is to protect the funding for sports and cultural events from sponsors on which they depend. The second reason is that, given the general policy of austerity and public spending cuts implemented in Ireland and Europe during the years under review (in response to the 2008–2009 financial crisis), all the main Irish political parties and the business sector were opposed to boosting public expenditure in general [52]. Therefore, sponsorship money became all the more important given the relative absence of public funds. The government and the business sector did not want to ban sponsorship and spend more public money to compensate for that loss.

Sixth, the media normalise alcohol drinking in Irish society. Indeed, one way to put in perspective the small coverage of the Bill and related measures is by comparing it with the much broader coverage provided to other alcohol topics, such as Guinness, Ireland’s national drink [49]. As Table 2 shows, there are seven times more articles that refer to Guinness than all pieces on the alcohol Bill, minimum pricing, and sponsorship (1889 vs. 266 pieces). Moreover, articles about Guinness are overwhelmingly positive or at least neutral in tone, with only approximately 2% negative. In other words, Guinness is normalised in Irish society. This goes some way toward cancelling out whatever positive coverage of public health measures appears in the media. Furthermore, Guinness is a narrow search: if one were to add to the equation other cultural references to alcohol, including other brands such as Jameson whiskey, the effect would be stronger.

Seventh, the media gloss over negative consequences associated with alcohol. In other words, what is omitted in the press is often more significant than what is printed. For example, a study found that the Irish media underreport the role of alcohol in confirmed alcohol-related deaths [53]. The authors document the “underreporting in Irish newspapers of the role of alcohol in traumatic and poisoning deaths” and mention that where “deaths were reported, the role played by alcohol was generally ignored” [53]. For instance, if a person dies because of an accident while inebriated after a night drinking at a bar, newspapers often simply report that the person had been “socialising”, without mentioning alcohol. The authors conclude that this “represents a missed opportunity to inform the public about the role of alcohol in these deaths” [53]. In the United States, the same findings have been made [54,55]. The proportion of news stories about car crashes, violent crimes, and other unintended injuries that mention the role of alcohol as a possible contributory factor is far lower than the actual proportion of events in which alcohol played a role. Those studies show that the media may favour the alcohol industry by simply not mentioning alcohol.

Eighth, policies that are priorities for the political and business establishment receive much larger amounts of positive media coverage than alcohol legislation. For instance, over the last five years, the Irish government has attempted to introduce a new tax on residential water consumption and install water meters for houses throughout the country. There has been public opposition to this policy but the media have remained overwhelmingly supportive of the water charges, which were part of the general policy of austerity following the financial crisis. Indeed, the same search parameters as those above (search: “water charge!” AT THE START) returned as many as 2007 articles on water charges: 498 in the *Irish Daily Mail*, 600 in the *Irish Times*, and 909 in the *Irish Independent*/*Sunday Independent*. The larger coverage cannot be explained by the greater financial importance of the proposed water tax or its significance as a policy issue for the national macroeconomy of the country compared to the alcohol Bill.

Another example is the large amount of positive media coverage for the strategy of budgetary austerity that was strongly supported by the establishment during the time period covered in this article. Empirical investigations limited to opinion pieces only in Irish newspapers found that between 2008 and 2013, 929 articles were published, with 58% supporting austerity but only 11% opposing it (the remainder were neutral) [27,52,56]. This is a much more significant media support than the coverage on the alcohol Bill, especially since it excludes the very large number of news pieces on the subject and considers only op-eds and editorials.

Ninth, the subdued media interest in alcohol issues is illustrated by the fact that there is not a single Irish journalist affected to covering alcohol issues, even though they are very significant for Irish society. There are, conversely, many journalists covering finance, party politics, and even sports and culture. It is thus difficult to maintain that the mass media is a strong advocate for alcohol-related public health issues.

Nevertheless, public health views related to alcohol do make their way into the media. The main reason for this is that the public health lobby is also a (small) part of elite circles, although with much less power than the corporate sector or the alcohol industry. Indeed, the medical professions and relatively progressive elements of government do have some power to influence policy and their views do receive coverage in the mass media. In fact, in Ireland, the last two decades have seen, for the first time, the emergence of a coherent public health “policy community” of experts and activists who have lobbied to implement public health-based alcohol policies. However, the drinks industry lobby has also been energised during the same time period. Ireland has thus witnessed increasingly adversarial relationships between the two camps. Overall, although public health advocates have prevailed over a few specific policy initiatives, the drinks lobby has prevailed over the general policy direction taken on alcohol matters [37]. The government has, overall, followed the interests of the drinks industry, having been reluctant to implement public health strategies to address alcohol problems [31,32,37].

## 5. Conclusions

This paper has presented an original critical political economy framework to conceptualise and understand the role of the media in alcohol-related issues. It conceives of media organisations as corporations that are part and parcel of the broader corporate world and thus sharing the latter’s values and interests. As a result, news outlets are not neutral, passive organisations that reflect the range of viewpoints in society; rather, they reflect the interests and viewpoints of political and economic elites and establishment institutions. Therefore, we should expect a significant amount of support for the alcohol industry in the mass media. This support may be active and explicit or passive and implicit. Moreover, we should expect some support for certain public health measures given the socioeconomic position of the medical professions and some progressive political figures, although their power is smaller.

This interpretation differs from others that conclude that the media have been supportive of alcohol-related public health approaches. For example, in the United Kingdom, Nicholls [57] argues that the normalisation of drinking in the news has declined and that public health advocates have successfully asserted their place in the media. In Australia, Azar et al. [9] find that alcohol use is portrayed more disapprovingly in recent years than a decade earlier. Similarly, Lemmens, et al. [58] maintain that US news media have given more prominence to public health in alcohol issues coverage. The situations in those countries may be inherently different than in Ireland. However, it would be interesting to revisit the above studies in light of this paper’s framework. The latter is applicable to media other than newspapers, including radio and television, as well as to content other than the news, such as movies, commercials, and popular culture representations.

In terms of recommendations for public health experts and advocates, this paper suggests that in order to increase media coverage of their perspectives, they should engage to a greater extent with the alternative media, which is less corporate, less commercial, and more diverse than the mass media. In the long-term, there is also a need to diversify the mass media and make news outlets less corporate in nature. True, engagement with the existing mass media may provide some important coverage and should not be dismissed altogether. However, the limits of this strategy should be recognised due to the media’s inherent tendency to resist, explicitly or implicitly, the implementation of strong public health measures.

Finally, two main limitations to this study should be noted. First, its conclusions are based on Ireland, but it would be interesting to investigate whether they hold in countries where the power of the alcohol industry and public health institutions is different. Ireland has a historically strong connection to the alcohol industry, but relatively weak public health institutions. Countries where the opposite is the case could necessitate different interpretations. Second, the recommendation to public health advocates to engage with alternative media is derived from the conceptual model of the mass media presented in the paper, but should be verified empirically. In other words, alternative media coverage of alcohol issues should be surveyed in order to examine precisely how they relate to alcohol issues. The alternative media in Ireland are so small that such a study would be difficult, but they are more vibrant in countries like the United Kingdom and the United States.

## Figures and Tables

**Table 1:** Number of articles in favour of and against alcohol policies, 2012–2017.

Policy	Pro	Against	Neutral	Total	% Pro	% Against	% Neutral
**Public Health (Alcohol) Bill**							
Irish Daily Mail	5	1	3	9	55.6	11.1	33.3
Irish Times	6	3	8	17	35.3	17.6	47.1
Irish/Sunday Independent	8	4	3	15	53.3	26.7	20.0
Average					46.3	19.5	34.1
**Minimum unit pricing**							
Irish Daily Mail	7	3	6	16	43.8	18.8	37.5
Irish Times	22	1	7	30	73.3	3.3	23.3
Irish/Sunday Independent	16	4	8	28	57.1	14.3	28.6
Average					60.8	10.8	28.4
**Ban on drinks industry sponsorship**							
Irish Daily Mail	7	10	12	29	24.1	34.5	41.4
Irish Times	24	13	21	58	41.4	22.4	36.2
Irish/Sunday Independent	22	19	23	64	34.4	29.7	35.9
Average					35.1	27.8	37.1
**Overall average**					44.0	21.8	34.2

**Table 2:** Number of articles mentioning Guinness, 2012–2017.

Newspaper	Total	Negative Articles	Negative Articles (%)
Irish Daily Mail	432	12	2.8
Irish Times	584	9	1.5
Irish/Sunday Independent	873	21	2.4
